# Editorial: Regulatory T cells in immune-mediated diseases

**DOI:** 10.3389/fimmu.2024.1537314

**Published:** 2024-12-23

**Authors:** Giang T. Tran, Nirupama Darshan Verma, Mark R. Nicolls, Bruce Milne Hall

**Affiliations:** ^1^ Immune Tolerance Laboratory, Faculty of Medicine, University of New South Wales (UNSW) Sydney, Sydney Australia an Ingham Institute, Liverpool, NSW, Australia; ^2^ Department of Medicine, Stanford University/VA Palo Alto, Sydney, CA, United States; ^3^ Department of Nephrology, Liverpool Health Service, Liverpool, NSW, Australia

**Keywords:** Treg - regulatory T cell, immune mediated, immune suppression, Foxp 3, pTreg cells

To ensure homeostasis, all biological systems exhibit both activating effects and regulatory mechanisms that counterbalance these effects. The discovery of regulatory and feedback mechanisms within immune responses has developed gradually over time. Thymic-derived T cells were first identified as inhibitory cells in 1970 (1) after the discovery of T cells by Miller in 1961 (2). Thymectomy in rodents shortly after birth led to the development of autoimmune responses (3), highlighting the regulatory role of the thymus. By the mid-1970s, research demonstrated that immune-mediated transplant tolerance, induced by neonatal infusion of allogeneic cells, could be transferred to a new host using enriched recirculating T cells (4). This observation initiated efforts to distinguish suppressor T cells from effector cells, an ongoing area of immunological investigation some of which is discussed in this Research Topic of papers and reviews.

During the 1970s and 1980s, research in murine models suggested the existence of CD8^+^ I-J^+^ T cells as suppressor cells. However, the inability to locate the I-J gene within the major histocompatibility complex (MHC) cast doubt on the existence of suppressor cells, leading to a substantial decline in investigations of suppressor T cells (5).

In a model of alloantigen-induced transplant tolerance in adult animals, it was observed that tolerance to specific alloantigen could be transferred by spleen and lymph node cells, as well as by enriched T cell preparations (6–9). Further characterization revealed that the tolerance-transferring T cells were CD4^+^ rather than CD8^+^. Unlike naive effector T cells, these CD4^+^ T cells did not rapidly recirculate from blood to lymph (10, 11). Further investigation demonstrated that these tolerance-inducing T cells lost suppressor function ex vivo within days, unless stimulated by a specific alloantigen and a cytokine-rich supernatant derived from Con A-activated T cells (12, 13). IL-2 was one cytokine that promoted the survival of alloantigen specific tolerance inducing T regulatory cells. This led to the observation that CD4^+^CD25^+^ T cells, which express the interleukin-2 (IL-2) receptor, were essential for the transfer of alloantigen-specific tolerance (14). At that time, expression of CD25 by activated T cells was considered a key marker of the rejection response and other. IL-2 was shown to partially sustain these tolerance mediating CD4^+^ cells, suggesting other cytokines were involved in the maintenance of functional T regulatory cells.

Early studies identified additional markers, including Class II MHC and CD45RC (14), on alloantigen-specific T cells. These markers continue to aid in the identification of activated CD4^+^CD25^+^ T cells in both humans and animal models.

Sakaguchi and colleagues later demonstrated that CD4^+^CD25^+^ T cells in mice could prevent autoimmunity induced by thymectomy performed 3-4 days after birth (15). These cells, now termed thymus-derived regulatory T cells (tTregs), are involved in regulating immune responses broadly. When activated by an antigen, they selectively activate antigen-specific regulatory T cells (Tregs) (16).

More than fifty years after thymocytes were first described as suppressors of immune responses, and over thirty-five years since CD4^+^CD25^+^ T cells were identified as suppressor cells, the complexity and heterogeneity of CD4^+^CD25^+^CD127^lo^Foxp3^+^ T cells are still not fully elucidated. Continued research in this field will deepen our understanding of Tregs, particularly their activation and function, providing insights into immune regulation and potential therapeutic applications.

The articles in this Research Topic covers a wide-ranging research and reviews of the phenotypes and unique characteristics of Treg as well as its roles in various human diseases.

A new hypothesis for beneficial effects of polyvalent immunoglobulin G (IgG) in immune tolerance is proposed by Victor and Nahm through integration of the pre-existing idiotypic network theory and Treg cell theory into an anti-idiotypic Treg cell theory. The theory is based on demonstrated *in vitro* and *in vivo* effects of polyvalent IgG. These include increased IL-10 expression *in vitro* on CD4^+^CD25^+^Treg, increased IL-10 expressing CD4^+^ cells in healthy donors, and increased CD4^+^Foxp3^+^cells in patients with atopic dermatitis associated with significant clinical improvement in patients. The proposed mechanism of IgG action involves multiple steps leading to IL-10 secretion by activated anti-idiotypic Treg through the presentation of immunogenic peptides generated from processing of IgG by dendritic cells. IL-10 in turn suppress Th2 cell response to allergens and autoimmune T cells response to self-antigens leading to IgG associated long-term clinical improvement in patients with allergic and autoimmune diseases. This theory needs validation by studying the detailed molecular mechanism underlying polyvalent IgG-induced Treg activation and examining its clinical usefulness.

Nirmala et al. conducted a detailed examination of various phenotypic markers to distinguish tTregs from pTregs and to identify activated tTregs. Liu et al., in contrast, characterized FoxP3^+^ Tregs and found that both resting (or naive) FoxP3^+^ Tregs and activated Tregs shared similar features, shedding light on the consistency of FoxP3 expression across Treg activation states.

Yilmazer et al. showed an unexpected high functional adaptability of peripheral Treg (pTreg) in absence of tTreg in mice model by selectively deleting either thymus-derived regulatory T cells (tTregs) or peripherally-induced regulatory T cells (pTregs). These pTregs acquire a highly activated suppressor phenotype and replenish the Treg cell pool. These observations contrast with the more established concept that tTregs are the primary mediators of self-tolerance. Overall, their study emphasized the important role of pTregs as mediators of self-tolerance and prompts further investigation of these cells for their potential use as a therapy for autoimmune diseases.

In a review on tissue resident regulatory T cells (TR-Tregs), Alvarez et al. provided insights about tissue residency program leading to generation of TR-Tregs. Inflammatory signals prior and during the migration of Tregs can alter the trajectory of these cells into tissues, leading them to adopt a helper T cell like phenotypes. Moreover, signals provided by other cytokines such as IL-12, IL-4 or IL-23 help them express T-bet^+^, GATA3^+^ or RORγ to express the appropriate Th chemokine receptor. This diverts T^+^ effector Treg to migrate to inflammation sites alongside conventional T cells. Mechanisms that help maintain the specific phenotypes have been discussed. We have previously shown that IL-2 and alloantigen activated tTregs express IL12Rβ2 and are stimulated by IL-12 to mediate potent donor specific suppression *in vivo* (17). Like pTregs, a better mechanistic understanding TR-Treg *in vivo* biology can ultimately guide their future development as a cell-based therapy.

In experimental lupus-prone mice, Rosenberger et al. demonstrated that Tregs play a critical role in inhibiting autoreactive effector T cells. Their study showed a strong inverse correlation between the levels of Tregs and antigen-specific autoreactive CD4^+^ T cells, emphasizing the direct role of Tregs in managing immune-mediated diseases.

In a comprehensive review, Xia et al. discussed the association between Tregs and cardiovascular diseases, presenting evidence from various studies that link Treg activity to cardiovascular health. They highlighted potential immunotherapies that may mitigate cardiovascular diseases by modulating Treg function.

Yilmaz et al. showed the role of Treg in pathogenesis of immune mediated liver disease, autoimmune liver disease (AILD) in scurfy mice and described features of disease pathogenesis. They confirmed that Treg deficiency is the key to spontaneous development of clinical, serological and immunopathological features of AILD. Wen et al. reviewed the role of T cells, mainly Th1/Th2 and Th17/Treg in pathogenesis of osteoarthritic (OA) and suggested future research directions for potentially new therapeutic strategies to include targeting and optimizing Th1/Th2 and Th17/Treg balance.

Zhang et al. investigated Treg populations in the blood of patients with sarcoidosis and found a significant correlation between sarcoidosis and a lower Treg population, alongside an elevated Th17.1 population. This imbalance suggests a dysregulation in immune cell populations in sarcoidosis patients.

Fukasawa et al. examined the efficacy of tildrakizumab, an IL-23 antibody, in patients with psoriasis. They found that clinical improvements were associated with enhanced Treg numbers and functionality, suggesting that tildrakizumab may exert therapeutic effects by promoting Treg-mediated immune regulation. Also, beneficial effects of supplements like glucosamine and chondroitin sulfate in repair and generation of chondrocytes need to be explored in detail. Vollmer et al. explored how leptin levels affect Treg proportions and functions in overweight individuals with allergies, finding that leptin influences Treg activity in this population.

Angelats and Santamaria provided a comprehensive analysis of the transcription factors that govern the development of type 1 regulatory T (Tr1) cells and the regulatory mechanisms that define their lineage, offering insights into the distinct biological pathways of Tr1 cells.

Overall these studies enhance our understanding of Treg biology, including their phenotypes, activation mechanisms, and functional pathways. Such studies provide significant promise for treating autoimmune and inflammatory diseases. Continued research will develop targeted immunotherapies that harness the unique suppressive functions of Tregs.
